# Aging heat treatment design for Haynes 282 made by wire-feed additive manufacturing using high-throughput experiments and interpretable machine learning

**DOI:** 10.1080/14686996.2024.2346067

**Published:** 2024-04-25

**Authors:** Xin Wang, Luis Fernando Ladinos Pizano, Soumya Sridar, Chantal Sudbrack, Wei Xiong

**Affiliations:** a Physical Metallurgy and Materials Design Laboratory, Department of Mechanical Engineering and Materials Science, University of Pittsburgh, Pittsburgh, Pennsylvania, USA; b National Energy Technology Laboratory, Albany, Oregon, USA

**Keywords:** Hot isotropic pressing, SHAP analysis, yield strength, heat treatment, Ni-based superalloy, machine learning, additive manufacturing

## Abstract

Wire-feed additive manufacturing (WFAM) produces superalloys with complex thermal cycles and unique microstructures, often requiring optimized heat treatments. To address this challenge, we present a hybrid approach that combines high-throughput experiments, precipitation simulation, and machine learning to design effective aging conditions for the WFAM Haynes 282 superalloy. Our results demonstrate that the γ’ radius is the critical microstructural feature for strengthening Haynes 282 during post-heat treatment compared with the matrix composition and γ’ volume fraction. New aging conditions at 770°C for 50 hours and 730°C for 200 hours were discovered based on the machine learning model and were applied to enhance yield strength, bringing it on par with the wrought counterpart. This approach has significant implications for future AM alloy production, enabling more efficient and effective heat treatment design to achieve desired properties.

## IMPACT STATEMENT

Our research tackles suboptimal properties in additively manufactured alloys with conventional heat treatment, using high-throughput experiments, CALPHAD (CALculation of PHAse Diagrams), and interpretable machine learning to effectively optimize heat treatments for WFAM (Wire-Feed Additive Manufacturing) Haynes 282 superalloy.

## Graphical abstract

**Figure:**
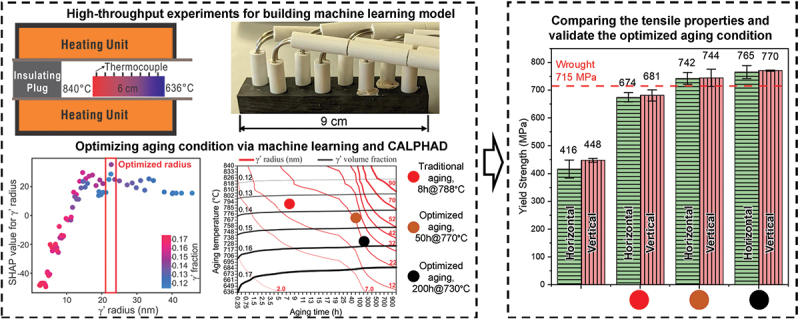

## 1. Introduction

Metal additive manufacturing (AM) produces components by depositing the material layer-by-layer and is capable of fabricating complex near-net shapes with reduced lead time [1,2]. Compared with other powder-based AM technologies, such as laser powder bed fusion (L-PBF), electron beam melting (EBM) and blown-powder directed energy deposition (DED), wire-feed additive manufacturing (WFAM) offers a higher deposition rate, cheaper feedstock, and it is suitable for high-volume production [3–5]. However, traditional post-processing techniques, such as thermomechanical processing and work hardening, cannot be applied to WFAM products due to their near-net shape. Moreover, heat treatment methods used for conventionally manufactured alloys may not always be suitable for AM parts and must be tailored based on the as-printed microstructure and target properties [6].

Haynes 282 is a nickel-based superalloy renowned for its exceptional formability, yield strength, and creep resistance, and it is widely used in applications ranging from aircraft engines and land-based gas turbines to advanced ultra-supercritical boilers [7–9]. The superior mechanical properties of Haynes 282 stem primarily from the presence of the γ’ (Ni_3_(Al, Ti)) phase within the grains, the solid solution strengthening, and carbides at the grain boundaries [10]. Proper heat treatment is crucial for obtaining the required precipitate type, size, and distribution. The conventional heat treatment of Haynes 282 entails a solution treatment (1121–1149°C), followed by two-step aging (1010°C for 2 h and 788°C for 8 h). The first step results in the precipitation of discrete M_23_C_6_ carbides along grain boundaries to increase the creep resistance, while the second step forms the appropriate amount of γ, which is higher than that of Haynes 263 (~12 mole.%) but lower than Waspaloy alloys (~24 mole.%) to improve strength, creep resistance and fabricability [11]. Several studies [8,12,13] have investigated the powder-based AM of Haynes 282 with conventional post-heat treatment, revealing that the yield strength differs depending on the additive manufacturing technique used. For instance, electron beam melting of Haynes 282 demonstrated a yield strength of 656 MPa and an elongation of 48% [12]. Conversely, powder-based DED resulted in a higher yield strength (710 MPa) and lower elongation (19%) [8]. Additionally, the yields strength of L-PBF manufactured alloy shows a yield strength greater than 940 MPa with elongation of 22.5% [13].

The relationship between aging heat treatment, microstructure, and the room-temperature yield strength of WFAM Haynes 282 remains an open area of investigation. Limited reports exist on WFAM Haynes 282, Zinke et al. [14] explored the influence of shielding gas on mechanical properties, Zhang et al. [3] did a systematically studied the carbide precipitates evolution, and Pizano et al. [15] found solution treatment temperature should increase from 1150°C to 1250°C to eliminate grain texture developed during WFAM. However, whether the conventional two-step heat treatment can achieve a yield strength comparable to desired yield strength of wrought counterparts (715 MPa) [16] remains unclear. Therefore, this study aims to use high-throughput experiments in conjunction with interpretable machine learning (ML) modeling [17] to optimize the second aging step of WFAM Haynes 282, improve room-temperature yield-strength, and explore its correlation with microstructure especially the γ’ precipitation.

## 2. Material and methods

### 2.1. Sample preparation

**Figure 1.**
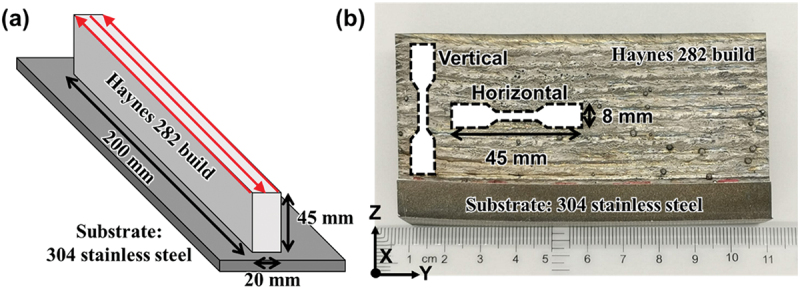
Printed sample illustration. (a) Schematic of the build (200 mm length, 20 mm width, and 45 mm height) and the scanning pattern used for printing. (b) Tensile bars extracted from the WFAM build in different orientations.

Wall-shaped structures (Figure 1(a)) were printed using Haynes® 282® wire of 1 mm diameter (wt.%: Ni-1.5Al-0.005B-0.06C-10Co-20Cr-1.5Fe-0.3Mn-8.5Mo-0.15Si-2.1Ti) with a WFAM system (Gefertec GmBH, Germany, GEFERTEC ARC 605 with Fronius TPS 400i PULSE power source) using a multitrack single bead printing strategy. A variety of processing parameters were optimized by adjusting conditions such as travel speed, wire feed rate, and power to ensure a stable melt pool. Finally, we determined the processing parameter as following: 950 mm/min torch travel speed and 6.5 m/min wire feed rate were maintained along with the 14 mm distance between the welding torch and the substrate. To generate a current of 140 A, an average voltage of 24 V was employed. The power (p) was 3.36 kW and the power density ($P_{d}$) is calculated using equation $P_{d}=\frac{4P}{\pi D^{2}}$ [18], which is 4280 W/mm^2^. Cronigon Ni10 (70% Ar + 30% He) gas was utilized as the shielding gas (20 L/min) for the torch to prevent oxidation of the build during the deposition process. Hot isostatic pressing (HIP, American Isostatic Presses Inc. Ohio, USA) was performed using the AIP6-45HMo furnace (American Isostatic Presses Inc.) with a heating rate of 15 K/min from room temperature to 1250°C, then hold at 1250°C and a pressure of 150 MPa for 2 hours followed by furnace cooling.

### 2.2. Microstructure characterization and mechanical property testing

Optical microscope (Carl Zeiss AG, Germany, ZEISS Axio Lab A1) was used for checking porosity. Microstructure characterization was performed after electrolytically etching the sample with chromic acid for 10 seconds using a voltage of 5 V and viewing it under a FEI Apreo secondary electron microscope (SEM, FEI, Thermo Fisher Scientific, USA). Grain structure analysis was performed using electron backscattered diffraction technique (EBSD, FEI, Thermo Fisher Scientific, USA) using EDAX Hikari EBSD system attached with the SEM and with further analysis using TSL-OIM(version8) software. Average microhardness was determined with a load of 300 grams and dwell time of 10 seconds from 40 measurements using an automated Vickers hardness tester (LECO Corporation, USA, AMH55 with LM310AT Microindenter). Tensile bars (MTS Systems Corporation, USA) of 45 mm length, 2 mm thickness, and 20 mm gauge length were extracted parallel and perpendicular to the build direction (YZ plane) for various heat treatment conditions (Figure 1(b)). Uniaxial tensile tests were performed using an MTS 880 universal testing machine with 100 kN capacity with a strain rate of 0.015 min^−1^, which is the standard strain rate listed in ASTM E8. The applied load was parallel to the build direction for the vertical samples and perpendicular to the build direction for the horizontal samples.

### 2.3. Precipitation simulation and machine learning modelling

The TC-PRISMA module of the Thermo-Calc Software, Sweden, [19] was used to predict γ’ phase fractions and radius with the TCNI11 and MOBNI5 databases for a range of aging conditions. For the interfacial energy, variations in composition and temperature can lead to different values. Consequently, we compared the measured radius with the calculated radius using various interfacial energy (0.01–0.05 J/m^2^) to determine the best fit. An interfacial energy of 0.023 J/m^2^ provides best fit. The grain aspect ratio was set to 0.39 based on EBSD measurement. The hyper-parameters of the gradient boosting algorithms for the ML that can provide accurate predictions and generalize well was determined by parameter grid search and evaluating the 3-fold cross-validation (CV) [20] metrics using Scikit-learn [21]. During this process, we divided the dataset into three subsets, and train model based on two subsets and evaluate model based on one dataset. After repeating 3 times, the root mean square error (RMSE) of the average performance was calculated and the set of model parameter yields lowest RMSE was chosen. Shapley Additive exPlanations (SHAP) [17] was utilized to interpret the predictions of the ML model.

## 3. Results and discussion

### 3.1. As-built and conventional heat-treated Haynes 282

**Figure 2.**
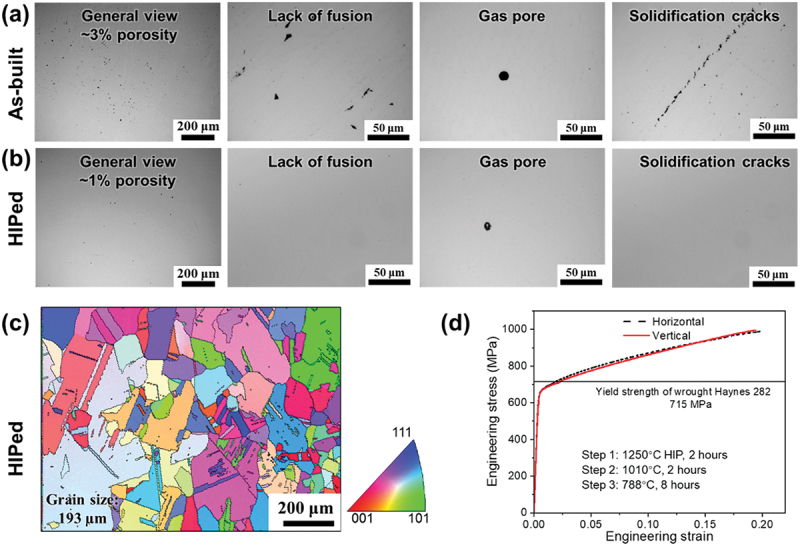
Characterization of WFAM haynes 282. (a) Optical micrographs of as-built haynes 282 alloy showing lack of fusion and gas pores with solidification cracks, (b) Optical micrograph of haynes 282 after HIP showing a reduced porosity (1%) with gas pores, (c) IPF map showing the grain structure of haynes 282 after HIP, (d) Engineering stress-strain curves after traditional second-aging at 788°C for 8 hours in a different orientation.

Based on Figure 2(a), the as-built WFAM Haynes 282 has a porosity of 3%, which negatively impacts its mechanical properties. As a result, the HIP treatment was performed at 1250°C for 2 hours at a pressure of 150 MPa followed by furnace cooling to reduce the porosity. The HIP temperature is higher than the conventional solution treatment temperature (1121–1149°C) to promote recrystallization of the as-printed microstructure and obtain an equiaxed grain structure [22]. As shown in Figure 2(b), most of the lack of fusion pores and solidification cracks were closed, with some remnant gas pores and the porosity reduced to 1%. The inverse pole figure (IPF) map in Figure 2(c) shows that the HIPed Haynes 282 has an average circular equivalent grain diameter of 193 μm, and the average grain size based on three EBSD measurements are 185 ± 25 μm, nearly double to fully heat-treated wrought Haynes 282 [11]. Based on Hall-Petch equation, larger grain size suggests a weaker grain boundary strengthening effect in WFAM HIPed Haynes 282. We measured the tensile properties of vertical and horizontal samples aged at 788°C for 8 hours, and it had a lower yield strength than the wrought counterparts (Figure 2(d)), necessitating the need to optimize the second aging step.

### 3.2. High-throughput experiments for building machine learning model

**Figure 3.**
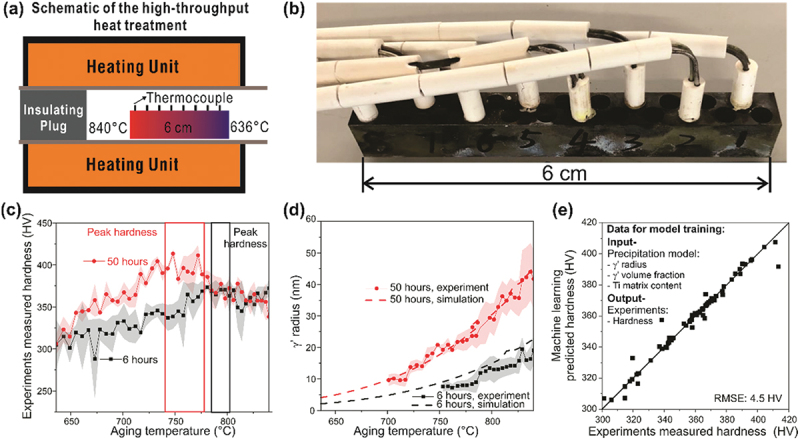
High-throughput experiments, precipitation simulation and ML for the design of the second-step aging. (a) Setup for the high-throughput treatment, (b) WFAM haynes 282 build with eight K-type thermal couple to monitor the temperature of different locations along the build, (c) Vickers hardness measured along the samples heat-treated for 6 and 50 hours, respectively. The points are the mean of the measured hardness, and the shaded area is the standard deviation, (d) The γ’ radius measured by SEM from the heat-treated sample where the points are the mean, and the shaded area is the standard deviation. It is not available for lower temperatures since the precipitate size is too small to be measured by SEM. The γ’ size calculated using the precipitation model is shown as the dash lines. (e) The comparison between the experimentally measured and the ML predicted hardness.

In order to determine a new aging condition that will make the WFAM Haynes 282 have comparable yield strength to the wrought counterparts and reveal the process-structure-property relationship, high-throughput gradient heat treatments were conducted with a setup shown in Figure 3(a,b) [23]. As Figure 3(a) shows, the sample bar was placed into the tube furnace with one end sealed with a thermal insulator and the other open to introduce the gradient in temperature along the tube ranging from 636 to 840°C at different locations of the sample bar. Further, eight equally spaced holes were drilled separately, and the thermocouples were fixed in 8 different locations to measure the temperature along the sample (Figure 3(b)). Throughout the heat treatment, temperature fluctuations with an error of ±0.75% was monitored.

Two WFAM samples were aged for 6 and 50 hours with the gradient temperature and subsequently quenched in ice water, where higher Vickers hardness was measured along the fully heat-treated samples, as shown in Figure 3(c). The thermally graded sample aged for 6 hours shows a peak hardness of 350 HV at 790°C, which is similar to the traditional second aging temperature; whereas the samples aged for 50 hours exhibits the highest hardness, close to 400 HV over a broad range of 740 to 770°C. This suggests that aging at a lower temperature for a longer period may result in greater strengthening effects.

The γ’ precipitates radius was analyzed using SEM and ImageJ software. TC-Prisma was used to calculate γ’ radius change with different aging times and temperatures and compared to measured values. Notably, Figure 3(d) demonstrates the high accuracy of TC-Prisma in predicting γ’ precipitation behavior over this temperature range. Our results show that the 6-hour sample achieves maximum hardness at a precipitate radius of approximately 12 nm, while the 50-hour sample gains the highest hardness when the radius is between 15–22 nm. These findings suggest that achieving the appropriate γ’ precipitate radius may further improve the material’s strength. Although a comparison of the experiments measured and model simulated γ’ volume fraction was desired, the etching of the Haynes 282 alloy after aging removed several surface layers up to a certain depth. Consequently, it became infeasible to distinctly observe the matrix because the γ’ phase from the underlying layers was also exposed, resulting in an apparent γ’ phase fraction of nearly 100% in every etched image. Therefore, the γ’ phase fraction was not determined from the SEM micrographs of the etched samples in this study. However, it is important to note that the calculated γ’ phase fraction for the fully aged alloy ranges from 0.14 to 0.18. This is closely aligned with the γ’ phase fraction that ranges from 0.13 to 0.19 reported by in other work [24,25], suggesting the calculation is accurate.

The ML model maps the relationship between the properties of interest and the material’s descriptors, i.e. model inputs [26]. Therefore, we can utilize a regression model to predict hardness using microstructural inputs, such as matrix composition and γ’ size and volume fraction that are the key structures to hardness and yield strength [27]. Figure 3(e) depicts the use of modeled material descriptors obtained from precipitation model to predict the hardness. During the modeling data curation, Vicker hardness outliers were excluded using ± 20 HV as a cutoff due to the large standard deviation observed in some hardness measurements for the same aging condition. Moreover, we performed the correlation study to find the linearly dependent features that have similar effects on hardness. Removing highly correlated features helps reduce model complexity and avoid overfitting. The matrix compositions show Pearson correlation coefficients higher than 0.99, and they are all highly correlated. Therefore, we excluded the matrix content of other alloying elements from our supervised machine learning model and retained only the γ’ volume fraction, γ’ radius, and Ti content as representative features. These values can be effectively predicted using a precipitation model, bypassing the need for costly experiments, and they significantly influence yield strength through precipitation and solid solution strengthening mechanisms. Conversely, we did not include grain size as model input features, which is contributing yield strength via grain boundary strengthening, due to the extensive experimental effort required for hundreds images of microstructure and its relatively minor contribution to yield strength in post-HIP samples with large grain sizes (185 μm). As Figure 3(e) shows, the prediction error is 4.5 HV, indicating that the model accurately captured the relationship between the input and output.

### 3.3. Interpretate machine learning model and design new heat treatment

**Figure 4.**
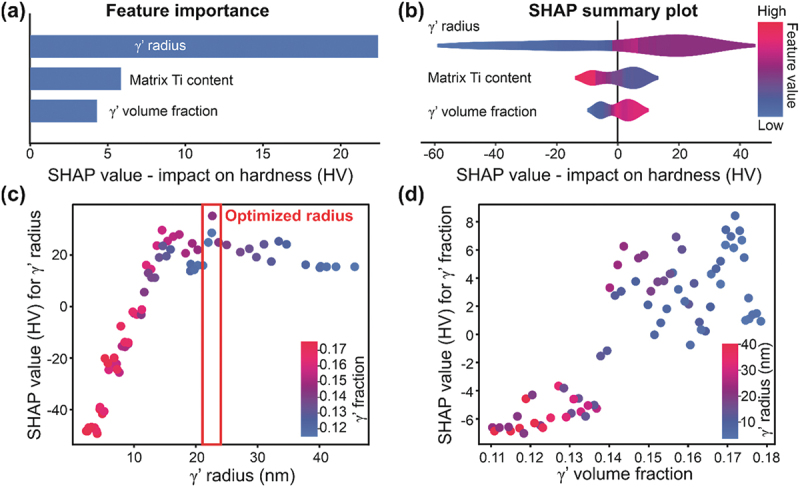
Interpreting the ML model using SHAP and heat treatment design. (a) Feature importance plot based on mean SHAP value, (b) SHAP summary plot, (c) SHAP plot for γ’ radius impact on the hardness, (d) SHAP plot for γ’ volume fraction impact on the hardness.

The ML model is generally considered as a black box data-driven model. However, we could adopt SHAP to extract helpful information from the data-driven model during the heat treatment design. The SHAP analysis applies Shapley values [28] from game theory to machine learning. Each feature is viewed as a player whose contribution to the model’s prediction task is assessed. SHAP values determine the impact of each feature by calculating the difference in the predicted outcome with and without the feature across all combinations of other features. Finally we get an additive explanation model where each feature’s contribution is measured relative to the average value for the whole training dataset [17]. Figure 4(a) presents the SHAP summary plot, which depicts the average impact of input feature on the hardness. The change of γ’ radius has the most significant impact on hardness, with an average effect close to 20 HV. In contrast, the Ti matrix composition and γ’ volume fraction have a much lower impact on the hardness (5 HV). Figure 4(b) shows that small γ’ radius (blue dots) results in a hardness reduction up to 60 HV compared to the dataset’s average hardness, whereas the impact is negligible if the radius is large (red dots). The hardness is highest when the radius is in the median (purple dots). Similarly, a higher Ti content in the matrix and lower γ’ volume fraction will lead to lower hardness/strength.

Figure 4(c) shows the impact of γ’ radius on hardness indicating that hardness will keep increasing as radius increases until a peak is reached when the radius is ~22 nm. Further increasing the γ’ radius will decrease the hardness. Moreover, for the same size, the red dots (higher volume fraction) always have higher SHAP values indicating increasing γ’ volume fraction is always beneficial for strength increment. Figure 4(d) reveals that the hardness is higher with a larger γ’ volume fraction. However, the y-axis scale is much smaller than Figure 4(c), confirming that the impact of γ’ volume fraction is less important than γ’ radius. Therefore, tuning γ’ radius appropriately is the key to strengthening Haynes 282.

To further verify the interpretable ML model output, we built a physics-based yield strength model study the impact of γ’ on the yield strength. Total yield strength σ can be calculated using the following equation:

$$\sigma =\sigma_{0}+\sigma_{\mathrm{grain}}+\sigma_{\mathrm{ss}}+\sigma_{p}$$

where $\sigma_{0}=69\mathrm{MPa}$ is the strength originating from lattice friction and dislocation density [29]. $\sigma_{\mathrm{grain}}$ = $\frac{k}{D^{0.5}}$ is the grain boundary strengthening, where *k* = 700 MPa/μm^0.5^ is the Hall-Petch coefficient [30] and *D* is the average grain size. $\sigma_{\mathrm{ss}}=\left(1-f\right){\left[\underset{i}{\sum }{\left(a_{i}c_{i}^{0.5}\right)}^{2}\right]}^{0.5}$ is the solid solution strengthening contribution, where *a*_*i*_ is the strengthening constant of element *i* in MPa/(atomic fraction^0.5^), listed as follows: a_Al_ = 225, a_Ti_ = 775, a_Co_ = 39.4, a_Cr_ = 337, a_Mo_ = 1015, a_Fe_ = 153, a_Mn_ = 448, a_Si_ = 275 [31], *c*_*i*_ is the matrix composition and *f* is the γ’ volume fraction calculated using TC-Prisma. The $\sigma_{p}$ is the precipitation strengthening effect, $\sigma_{p}=\min\left(\sigma_{p\_\mathrm{orowan}},\sigma_{p\_\mathrm{shear}}\right)$, where $\sigma_{p\_\mathrm{orowan}}$, and $\sigma_{p\_\mathrm{shear}}$ are Orowan looping and shear strengthening effect, respectively. The Orowan mechanism can be calculated by $\sigma_{p\_\mathrm{orowan}}=\frac{3\mathrm{M\mu b}}{2L}$, where *M* = 3 is the Taylor factor [32], *μ* = 82 GPa and *b* = 0.248 nm are the shear modulus and burgers vector, respectively [29]; $L={\left(\frac{2\pi }{3f}\right)}^{0.5}r$ is the mean particle spacing of γ’ precipitates, and *r* is the γ’ radius calculated using TC-Prisma. For the shear mechanism, we adopted the model developed by Galindo-Nava et al. [30] since it presents good agreement with experimental results [30] and significant improvements over the traditional weak and strong pair-coupling models by addressing their inherent limitations [33,34]. For instance, conventional weak pair-coupling models often inaccurately predict a ‘negative’ strengthening effect in smaller particles with lower volume fractions by overestimating the pinning effects on trailing dislocations [35]. Unlike these models, which deal with extreme dislocation configurations and incorrectly assume a smooth transition from weak to strong pair-coupling at maximum strength, the unified model offers a more precise description. It refines the dislocation configuration for weak pair-coupling and determines maximum strength occurs when the dislocation bowing angle is null [36], rather than at the convergence point of weak and strong pair-coupling assumed by conventional models. Moreover, it introduces an intermediate configuration that effectively bridges weak and strong pair-coupling, providing a more accurate equilibrium force balance and enhancing predictions of precipitation strengthening effects. The anti-phase boundary energy of the γ’ phase is 0.18 J/m^2^ [29].

**Figure 5.**
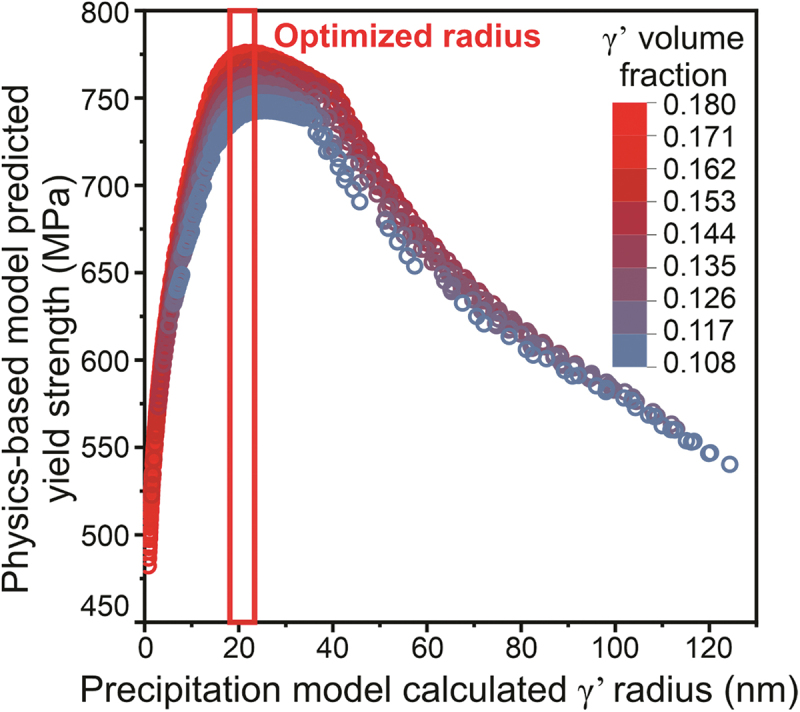
Precipitation model calculated γ’ radius versus the physics-based yield strength model predicted yield strength for different aging conditions.

Figure 5 demonstrates that the results from the yield strength model are consistent with those from the ML model. An increase in γ’ volume fraction (red dots) results in a slight increase in yield strength, while γ’ size has a much greater impact, with the lowest and highest yield strength being 450 and 770 MPa, respectively. Moreover, the yield strength also reaches a maximum when the γ’ radius is between 21–25 nm. Compared to the ML model, the physics-based yield strength model usually requires additional parameters and a well-established theoretical model. Conversely, the ML model needs a database from literature or high-throughput experiments, showing the capability for exploring post-heat treatment and learning the process-structure-property relationship rapidly for new alloys or manufacturing process.

**Figure 6.**
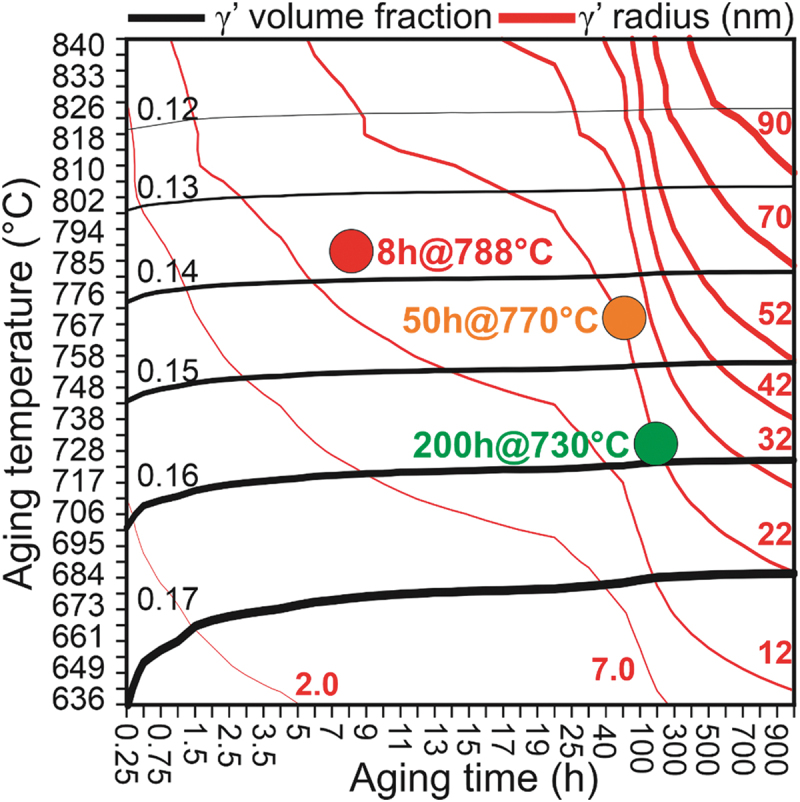
Heat treatment condition determination based on contour plot of the precipitation model calculated γ’ size and volume fraction. The simulation aging time ranges from 0.25 to 1000 hours and temperature between 636 and 840°C.

Figure 6 shows precipitation model predicted γ’ radius and volume fraction for range of aging temperatures (636–840°C) and times (0.25–1000 hours), where the solid red and black lines indicate constant γ’ radius and volume fraction values, respectively. The various aging conditions discussed are marked, and plot identifies the heat treatment that leads to a γ’ radius of 22 nm (Figure 6). The traditional second-step aging condition is red with a γ’ radius of 12 nm and a volume fraction of 0.14 was used as a reference. Two new aging conditions with a radius of 22 nm and volume fraction close to 0.15 and 0.16 were chosen, which are 50 hours at 770°C and 200 hours at 730°C, respectively.

### 3.4. Design validation and discussion

**Figure 7.**
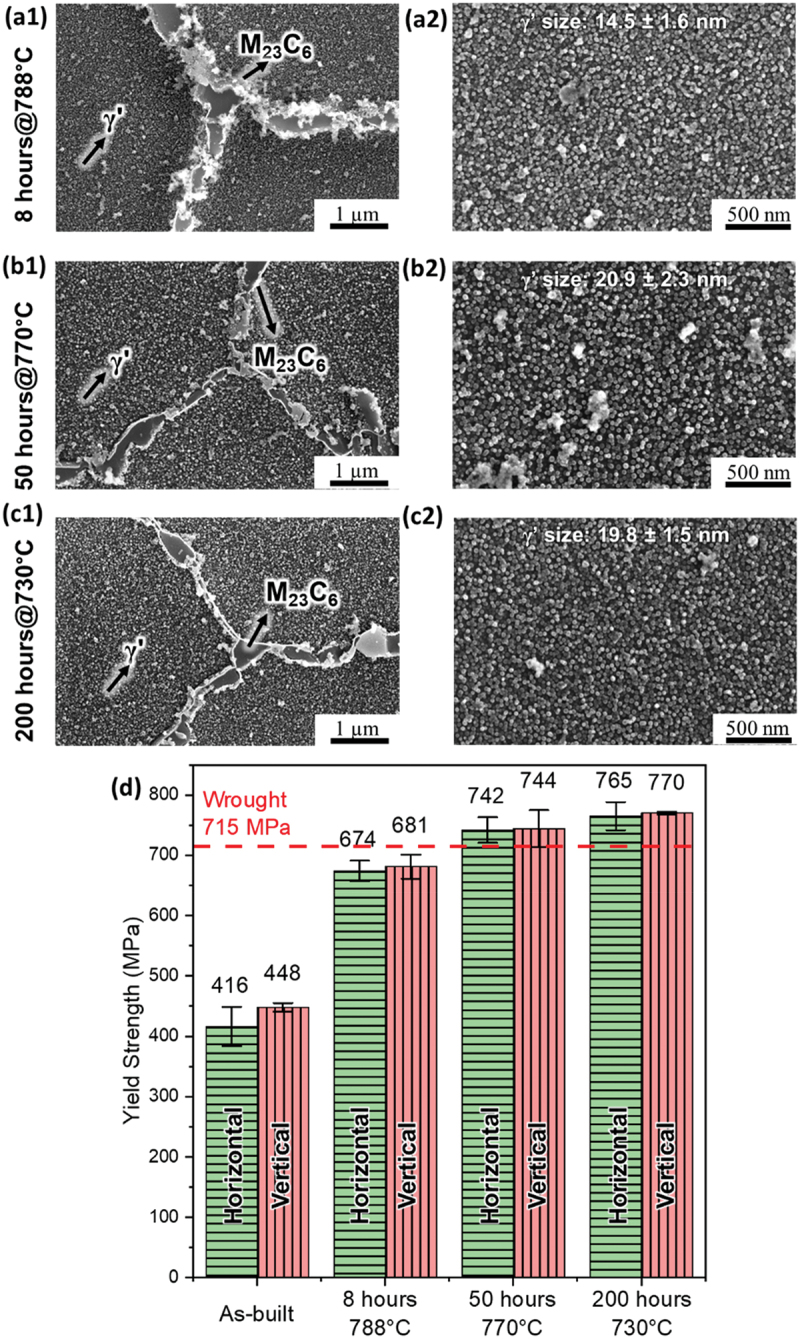
Yield strength and microstructure of different second aging heat treatments in different orientations. SEM image of samples (a) Aged at 788°C for 8 hours, (b) Aged at 770°C for 50 hours, (c) Aged at 730°C for 200 hours, where (1) is the image at lower magnification showing the grain boundary carbides and γ’ in matrix and (2) is the image at higher magnification with the measured γ’ radius, and (d) Bar plot summarizing the yield strength of WFAM haynes 282 in different conditions and orientations and its comparison with wrought haynes 282 alloy.

Figure 7(a–c) show the resulting microstructure of WFAM Haynes 282 for the three different second aging conditions: 788°C for 8 hours, 770°C for 50 hours and 730°C for 200 hours. The resolution of SEM was sufficient to detect the γ’ precipitates within the grains, and the measured γ’ precipitate radius values are very close to the predicted values, validating that precipitation model can reliably predict the precipitation kinetics of γ’. Moreover, there are discrete M_23_C_6_ decorate the grain boundary with no film formed for all optimized aging, which is beneficial for creep resistance [37]. Figure 7(d) summarizes the yield strength after different heat treatments, and the red dashed line denotes the yield strength of wrought Haynes 282. The yield strength of as-built and traditional heat treatment at 788°C for 8 hours did not match their wrought counterpart. However, after aging at 770°C for 50 hours and at 730°C for 200 hours, the yield strength surpassed the target. This indicates that ML-guided aging design can enhance the yield strength of WFAM Haynes 282, which proves the successfulness of this design.

Certainly, a more extensive investigation assessing different critical properties, such as the creep resistance and ductility, is necessary to fulfill the industrial application requirements. However, such work will requires more extensive experiments and implementation of multi-objective optimization techniques [38]. Instead of establishing optimal heat treatment parameters that satisfy all properties, this study aims at demonstrating that the combination high-throughput experimentation, precipitation model, and interpretable machine learning technique can rapidly enhance our understanding of how heat treatment parameters affect specific properties of additive manufactured parts and to assist in the design process. This is of paramount importance as the microstructures in additive manufactured alloys vary significantly based on the manufacturing techniques and printing parameters [39], which may requires swiftly adjusting heat treatment conditions for an alloy manufactured with various conditions.

## 4. Conclusions

In summary, we found that the WFAM technique is capable of efficiently producing large components, however, post-heat treatment optimization is required since the yield strength of WFAM manufactured alloy after conventional aging treatment is only 674 MPa, which is lower than the yield strength 715 MPa of wrought counterpart. To address this issue, a database was generated using high-throughput experiments and precipitation model. This database was utilized by a ML model to map the process-structure-property relationships along with SHAP to guide the design of the second aging heat treatment for WFAM Haynes 282. The key findings are listed as below:

- SHAP analysis revealed that the γ’ precipitate size significantly influences yield strength – four times more than matrix composition and γ’ volume fraction. Optimal strengthening occurs at a γ’ size of 22 nm.
- Aging the alloy at 770°C for 50 hours achieves the ideal γ’ precipitate size, enhancing the yield strength to 742 MPa, surpassing that of the wrought alloy.
- The combination of high-throughput experiments, modeling, and ML serves as a new paradigm to accelerate the development of post-heat treatment for AM alloys.

## Data Availability

Data will be made available on request.

## Disclaimer

This report was prepared as an account of work sponsored by an agency of the United States Government. Neither the United States Government nor any agency thereof, nor any of their employees, makes any warranty, express or implied, or assumes any legal liability or responsibility for the accuracy, completeness, or usefulness of any information, apparatus, product, or process disclosed, or represents that its use would not infringe privately owned rights. Reference herein to any specific commercial product, process, or service by trade name, trademark, manufacturer, or otherwise does not necessarily constitute or imply its endorsement, recommendation, or favoring by the United States Government or any agency thereof. The views and opinions of authors expressed herein do not necessarily state or reflect those of the United States Government or any agency thereof.
